# Responses of absolute and specific enzyme activity to consecutive application of composted sewage sludge in a Fluventic Ustochrept

**DOI:** 10.1371/journal.pone.0177796

**Published:** 2017-05-17

**Authors:** Xiao Liu, Kangli Guo, Lin Huang, Zhengyu Ji, Huimin Jiang, Hu Li, Jianfeng Zhang

**Affiliations:** 1 Institute of Agricultural Resources and Regional Planning, Chinese Academy of Agriculture Sciences, Beijing, China; 2 Sewage Purification Co., Ltd., Zhengzhou, China; RMIT University, AUSTRALIA

## Abstract

Composted sewage sludge (CS) is considered a rich source of soil nutrients and significantly affects the physical, chemical, and biological characteristics of soil, but its effect on specific enzyme activity in soil is disregarded. The present experiment examined the absolute and specific enzyme activity of the enzymes involved in carbon, nitrogen, and phosphorus cycles, the diversity of soil microbial functions, and soil community composition in a Fluventic Ustochrept under a maize—wheat rotation system in North China during 2012–2015. Application of CS led to increase in MBC and in its ratio to both total organic carbon (TOC) and microbial biomass nitrogen (MBN). Absolute enzyme activity, except that of phosphatase, increased in CS-treated soils, whereas specific activity of all the enzymes declined, especially at the highest dose of CS (45 t ha^−1^). The diversity of soil microbial community also increased in CS-treated soils, whereas its functional diversity declined at higher doses of CS owing to the lowered specific enzyme activity. These changes indicate that CS application induced the domination of microorganisms that are not metabolically active and those that use resources more efficiently, namely fungi. Redundancy analysis showed that fundamental alterations in soil enzyme activity depend on soil pH. Soil specific enzyme activity is affected more than absolute enzyme activity by changes in soil properties, especially soil microbial activity and composition of soil microflora (as judged by the following ratios: MBC/TOC, MBC/MBN, and TOC/LOC, that is labile organic carbon) through the Pearson Correlation Coefficient. Specific enzyme activity is thus a more accurate parameter than absolute enzyme activity for monitoring the effect of adding CS on the activities and structure of soil microbial community.

## Introduction

Sewage sludge is widely regarded as a useful soil amendment for improving the biological and physicochemical properties of soil—especially sandy soils—by providing nutrients and organic material [1–2]. Such use of sewage sludge not only improves soil quality but also offers a way to dispose of sewage sludge—a potential environmental pollutant—in large quantities [3]. Sewage sludge is high in metals, organic pollutants, and pathogenic organisms [4] and therefore requires some treatment before use. The organic matter in sewage sludge is biodegradable and can be transformed into a humus-like product, which is called composted sewage sludge (CS), under aerobic conditions by composting [5]. However, the metals in CS are a potential environmental hazard [6], and such trade-offs in using CS need to be evaluated [7].

Addition of CS can not only enhance the total organic carbon (TOC) but also change its active components, such as labile organic carbon (LOC) and microbial biomass carbon (MBC). One of the properties most sensitive to these changes in the forms of carbon in soil is the activity of enzymes involved in the cycling and availability of nutrients, degradation and synthesis of soil organic matter, and biodegradation of toxic organic pollutants. This sensitivity makes soil enzyme activity a good indicator of soil quality [8]. Enzyme activity can show the activity of microorganism, rates of decay, and utilizability of substrates by plants and microbes [9]. The activity of cellobiohydrolase (CBH), β-1,4-glucosidase (βG), α-1,4-glucosidase (αG), β-xylosidase (βX), β-1,4-N-acetylglucosaminidase (NAG), acid (alkaline) phosphatase (AP), and urease (UA) regulates the discharge of bioavailable nutrients from organic carbon (C), nitrogen (N), and phosphorus (P) [10–12] and often benefits from addition of organic materials [13]. Absolute enzyme activity and its stability in soil are also influenced by soil pH, nutrients, microbial biomass, C pool, and microbial community composition [14–15]; all these factors, in turn, are influenced by organic matter, nutrients, and microbes in CS. However, if applied in excess, CS can also lower soil quality and productivity [4, 5]. The response of absolute enzyme activity to additions of CS—especially its influence on enzymes involved in C, N, and P cycles in sandy soils—is yet to be fully understood. Secondly, not all soil enzymes are affected the same way, which is why the sensitivity of different soil enzymes to CS needs to be examined further [16].

Compared to absolute enzyme activity, specific enzyme activity can be used to normalize the differences in MBC between different treatments and is a more accurate measure for comparing the extent to which different soil treatments affect the functioning of soil microbial community. Specific enzyme activity also gives information on the metabolic state of alterations in the microbial community, enzyme efficiency, and ecology-related information about activities of the immobilized extracellular enzymes [17]. In addition, specific enzyme activity can also serve as a functional indicator with direct ecological impacts and therefore could be more readily used as an indicator of alterations in soil microbial community composition [18].

Studies on specific enzyme activity, however, have focused more often on the differences in land use [17, 19–20] and to date, little information is available on how specific enzyme activity is affected by continued application of CS. Specific enzyme activity may be more sensitive than absolute enzyme activity to changes in soil properties [18, 20] and can therefore be used to monitor the impacts of CS more closely and to avoid the ill effects of its excessive application. It is important to study the relationship between specific enzyme activity and soil microbial community composition in different agro-ecosystems. More specifically, we need to find out the impacts of different doses of CS on specific enzyme activity and its relationship with soil microbial community composition. It is against this background that the present experiment sought to (1) assess the influence of different doses of CS on absolute and specific enzyme activity; (2) elucidate the mechanisms of changes in soil absolute and specific enzyme activity; (3) study the sensitivity of soil functional diversity and microbial community composition to specific enzyme activity; and (4) identify the main environment factors that mediate the effects of adding CS on enzyme activity using various methods of statistical analysis.

## Materials and methods

### Sewage sludge

Samples of CS were collected from Zhengzhou municipal wastewater treatment plant in Henan province, China (34.76° N, 113.91° E). A mix containing equal amounts (by weight) of peanut shells and maize stalks was added to the sludge in a ratio of 1:5 (w/w) and the resultant substrate was inoculated with a consortium of *Bacillus subtilis*, *Aspergillus niger*, and *Sporotrichum thermophile* to produce a commercial-quality product through aerobic fermentation and high-temperature composting within three weeks. The main properties of CS are listed in Table 1. The contents of main heavy metals were below the limits stipulated in the Control Standard of Pollutants in Agricultural Sludge (GB/T 24600–2009 China): the sludge was therefore fit for its intended use as a soil amendment.

**Table 1:** Major physicochemical properties of soil (initial values) and of composted sewage sludge.

	Soil	Composted sewage sludge	The maximum capacity set by GB/T 24600–2009, China
pH	8.42	8.05	
Available P (mg kg^-1^)	13.20	—	
Total P (g kg^-1^)	—	9.86	
Available K (mg kg^-1^)	40.32	—	
Total K (g kg^-1^)	—	13.90	
Total N (g kg^-1^)	0.44	17.60	
Total organic C (g kg^-1^)	7.05	130.64	
Dissolved organic C (mg kg^-1^)	——	2927	
Labile organic C (g kg^-1^)	——	15.8	
C/N	16	7.43	
Available nitrogen (mg kg^-1^)	20.33	——	
Total Cr (mg kg^-1^)	28.71	200.49	1000
Total Ni (mg kg^-1^)	12.31	55.25	200
Total Cu (mg kg^-1^)	9.77	164.35	1500
Total Zn (mg kg^-1^)	22.90	338.24	4000
Total Cd (mg kg^-1^)	0.14	1.31	20
Total Pd (mg kg^-1^)	13.95	22.25	1000

### Experimental area

The experimental area was part of the experimental plot of the Kaifeng Academy of Agriculture and Forestry in Henan province, China (34.77° N, 114.27° E). The region has a subarctic climate (according to the Koppen Climate Classification) with the following annual values between 2012–2015: 2012, mean temperature, 15.38°C, rainfall, 467.1 mm; 2013, mean temperature, 15.66°C, rainfall, 335.3 mm; 2014, mean temperature, 15.87°C, rainfall, 508.1 mm; 2015, mean temperature, 15.42°C, rainfall 583.6 mm. The type of soil used in the experiment was Fluventic Ustochrept. The major characteristics of topsoil (0–20cm) are given in Table 1.

### Experimental design

The experiment, conducted from October 2012 to October 2015, consisted of four treatments, each with three replications, and was carried out as a completely randomized block design comprising 12 plots (each 5 m^2^, 2 m × 2.5 m) separated by cement walls. The plots were under a maize—wheat rotation. The four treatments, with varying doses of CS and a control, were as follows: (1) CK (‘check’, or control), inorganic fertilizers alone; (2) CS1, inorganic fertilizers and 15 t ha^−1^ of CS; (3) CS2, inorganic fertilizers and 30 t ha^−1^ of CS; and (4) CS3, inorganic fertilizers and 45 t ha^−1^ of CS. During each growing season of maize and wheat, all the plots received a uniform dose of inorganic fertilizers (225 kg ha^−1^ of N as urea, 86 kg ha^−1^ of P_2_O_5_ as mono ammonium phosphate, and 113 kg ha^−1^ of K_2_O as potassium chloride). Both CS and the inorganic fertilizers were applied as a basal dose by broadcasting and immediately incorporated into the top layer of the soil (0–20 cm depth) by ploughing. The application of CS and fertilizers preceded sowing.

### Experimental methods

#### Soil collection and analysis

After the harvest of maize, soil samples were gathered from each experimental plot in October 2015. Soil cores (each 6 cm in diameter) were taken from the top layer (0–20 cm) from five spots selected at random in each plot to constitute an integrated sample. Soil samples (dry) were sifted using a 2-mm mesh sieve and stored at room temperature until the chemical analyses were performed. Soil samples (fresh) were also passed through a 2-mm mesh sieve, and plant roots were removed. The fresh samples were stored at -20°C for analysis of enzyme activity and microbial community composition.

#### Chemical analysis

Soil organic matter was measured using the method of dichromate oxidation. Soil pH was measured with a glass electrode (PE-10, Sartorius, Gottingen, Germany) dipped in a soil suspension prepared by mixing 1 part of soil with 2.5 parts of water (w/v). Available P was measured by the Olsen method [21]. Soil ammonium and nitrate nitrogen were extracted by 1 mol·L^−1^ KCl by mixing 1 part of soil with 10 parts of water (w/v) and the extracts were analysed using a flow analyzer (Seal Autoanalyzer 3, Norderstedt, Germany).

Soil microbial biomass C and N were determined by the method of chloroform fumigation—extraction [22]. To determine the LOC, the samples (1.5 g each) were oxidized in 333 mmol·L^−1^ KMnO_4_ for 24 h at 25°C and then centrifuged for 5 min at 2000 rpm; the supernatant was diluted 250 times with deionized water; and LOC was quantified by colorimetric analysis at 565 nm [23].

To analyse the potentially toxic elements, dried soil samples (0.5 g each) were treated with 10 mL HCl at 100°C for 1 h and then with a mix containing 5 mL of HF, 5 mL HNO_3_, and 3 mL HClO_4_ at 200°C until the black residue disappeared. After cooling, the extracts were made up to 50 mL with ultrapure water. The contents of heavy metals—Cu, Zn, Cd, Cr, Ni and Pb—were determined using inductively coupled plasma mass spectrometry (ICP-MS) [24]. Blanks, control samples, and certified reference materials (GBW07446, GSS-17, sandy soil, CHN) were used for quality control. The recovery of standards ranged from 85% to 100%.

#### Enzyme activity

The potential activity of six enzymes (Table 2) was analysed using the microplate fluorometric protocol [25].

**Table 2:** Enzymes with corresponding commission number (EC), corresponding substrate, and the abbreviation used in this study.

Enzyme	Abbreviation	Substrate	EC
phosphatase	Pho	4-MUBa-phosphate	3.1.3.1
β-Glucosidase	βG	4-MUB- glucosidase	3.2.1.21
Cellobiohydrolase	CBH	4-MUB-β-D-cellobioside	3.2.1.91
N-Acetyl-glucosaminidase	NAG	4-MUB-N-acetyl-β-D-glucosaminide	3.2.1.30
β-Xylosidase	βX	4-MUB-β-D-xyloside	3.2.1.37
α-Glucosidase	αG	4-MUB-α-D-glucoside	3.2.1.20

Urease activity (UA) was measured as follows: to 5 g of soil sample was added 1 mL of methylbenzene and, after 15 min, the sample was mixed with 10 mL of 10% urea solution and 20 mL of citrate buffer (pH 6.7). The mixture was filtered after incubation at 37°C for 24 h, and 4 mL of sodium phenolate solution and 3 mL of sodium hypochlorite solution were added to 3 mL of the filtrate. After 20 min, the solution was diluted with distilled water to make up the final volume to 50 mL, and the released ammonium was assayed colorimetrically at 578 nm.

Catalase activity (CA) was determined as follows: to 2 g of soil sample were added 5 mL of 0.3% H_2_O_2_ and 40 mL distilled water and, after shaking for 20 min, the suspension was titrated with 0.1 N KMnO_4_ [26].

Soil microbial functional diversity was calculated from the activities of eight enzymes using Shannon’s diversity index [9] as follows: $H^{\prime }=-\Sigma_{i=1}^{5}PiLnPi$ where *Pi* is the ratio of the activity of each enzyme to the sum of activities of all enzymes.

#### Microbial community diversity

DNA was extracted using the FastDNA Spin Kit for Soil (MP Biomedicals LLC, Santa Ana, CA, USA) and subjected to polymerase chain reaction. The pyrosequencing data were analysed using QIIME, a software package. Because of the wide range of sample sizes among treatments, the number of sequences was standardized for all samples to obtain unbiased estimates of the diversity. The minimum number of reads, corresponding to the CK sample, was 39,530. Therefore, a number of reads were randomly selected from each sample to yield 39,530 reads in the normalized data set, and microbial community diversity was estimated by Mothur, a software package.

#### Statistical analysis

Statistical differences were analysed using SPSS 19.1 with one-way analysis of variance, and Fisher’s LSD test (α = 0.05) was performed to separate mean values. The correlations between absolute and specific enzyme activities and soil physicochemical properties were analysed with Pearson’s correlation analysis. Principal component analysis (PCA) was used to compare bacterial community structure, based on operational taxonomic units (OTUs), and also to compare absolute and specific enzyme activities across all the samples. Redundancy analysis (RDA) was carried out using CANCO 5.0 to analyse multiple correlations between environmental factors and enzyme activity. A manual forward-selection procedure with a Monte Carlo test (499 permutations) was conducted to measure the significance of environmental factors.

## Results

### Soil physicochemical properties

Application of CS lowered the soil pH significantly and the dose of CS was negatively correlated to the pH; CS3 lowered the pH the most, by 1.83 (Table 3). The amended soil also showed higher levels of TOC, TN, available P, and NO_3_^−^, and the increase was more marked at higher doses of CS, whereas NH_4_^+^ increased significantly only in CS2 and CS3 (the difference between these two treatments was not significant).

**Table 3:** Major physicochemical properties of soil samples under different treatments.

	pH	TOC (g kg^-1^)	TN (g kg^-1^)	NH_4_^+^ (mg kg^-1^)	NO_3_^−^ (mg kg^-1^)	AP (mg kg^-1^)	MBC (mg kg^-1^)	LOC (g kg^-1^)	MBC/TOC	LOC/TOC(%)	MBC / MBN
CK	8.90±0.09a	5.52±0.36d	0.56±0.04d	1.31±0.13c	3.05±0.35c	14.4±2.24d	89.37±22.09d	1.71±0.06d	1.62±0.02b	29.23±0.84c	4.04±0.08b
CS1	8.56±0.11b	8.40±0.43c	0.99±0.03c	1.15±0.16c	5.19±0.11b	45.71±4.28c	164.45±32.55c	3.58±0.04c	1.96±0.01a	42.81±2.04a	5.06±0.13a
CS2	8.40±0.06b	11.15±0.24b	1.40±0.02b	1.78±0.13a	7.68±0.58a	73.6±3.18b	215.93±44.03b	4.12±0.13b	1.94±0.06a	36.95±0.33b	4.90±0.24a
CS3	8.07±0.01c	14.74±0.74a	1.55±0.05a	1.48±0.02ab	8.23±0.54a	84.90±3.11a	257.45±57.95a	4.88±0.06a	1.75±0.04b	33.25±1.78bc	4.47±0.28ab

Values are mean ± standard deviation (N = 3). Different letters indicate significant differences in the same column (*P* <0.05, Fisher’s LSD test). Treatments: CS1 (CS at 15 t ha^−1^ + inorganic fertilizers), CS2 (CS at 30 t ha^−1^ + inorganic fertilizers), CS3 (CS at 45 t ha^−1^ + inorganic fertilizers), CK (inorganic fertilizers only). CS: composted sewage sludge, TOC: total organic carbon, TN: total nitrogen, AP: available phosphorus, MBC: microbial biomass carbon, MBN: microbial biomass nitrogen. These abbreviation are used in all the figures and tables.

Adding CS also increased the two active fractions of organic carbon significantly: MBC by 84.00%–188.07% and LOC by 26.26%–58.03% (Table 3). As for the ratios, they were both significantly higher in CS1 and CS2 than in CK: MBC/TOC by 20.77% in CS1 and 19.45% in CS2, and LOC/TOC by 46.48% in CS1 and 26.49% in CS2. However, the ratios did not differ significantly between CS3 and CK. There was an increasing trend in MBC/MBN but the increase was significant only in CS1 and CS2.

### Absolute and specific enzyme activity

**Fig 1:**
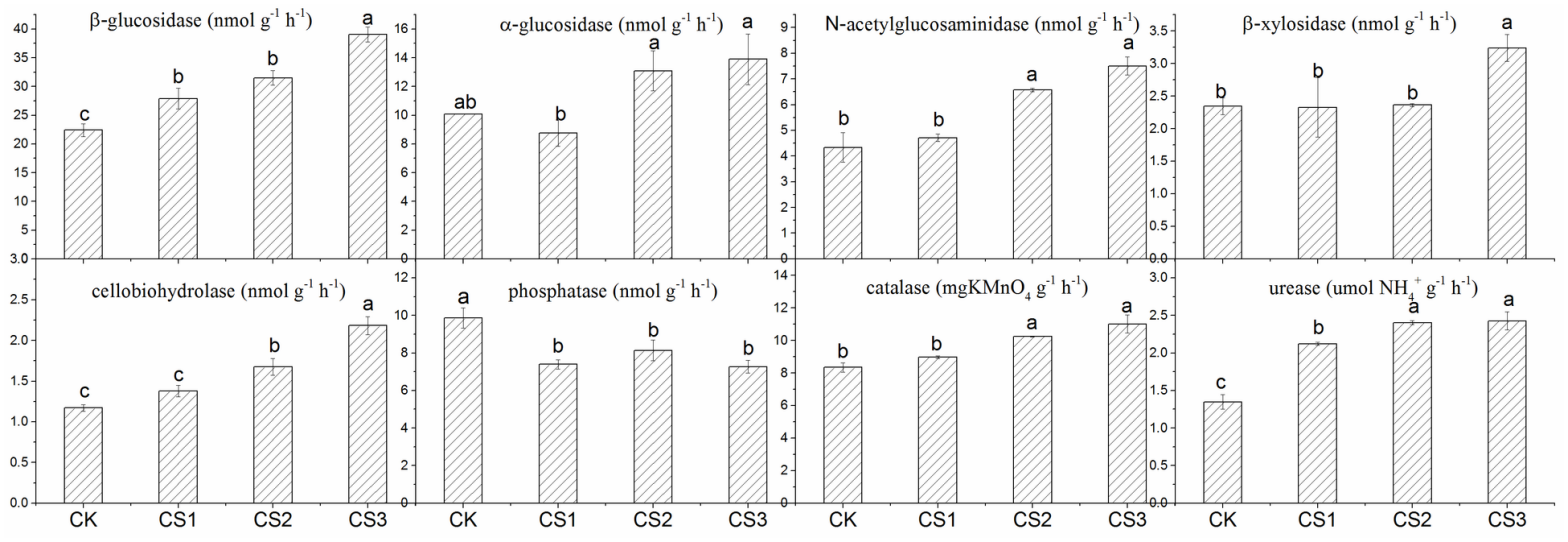
Effect of application of composted sewage sludge (CS) on absolute enzyme activity. Pho: phosphatase, CA: catalase, βG: β-glucosidase, CBH: cellobiohydrolase, NAG: N-acetylglucosaminidase, βX: β-xylosidase, αG: α-glucosidase, UA: urease. These abbreviation are used in all the figures and tables.

**Fig 2:**
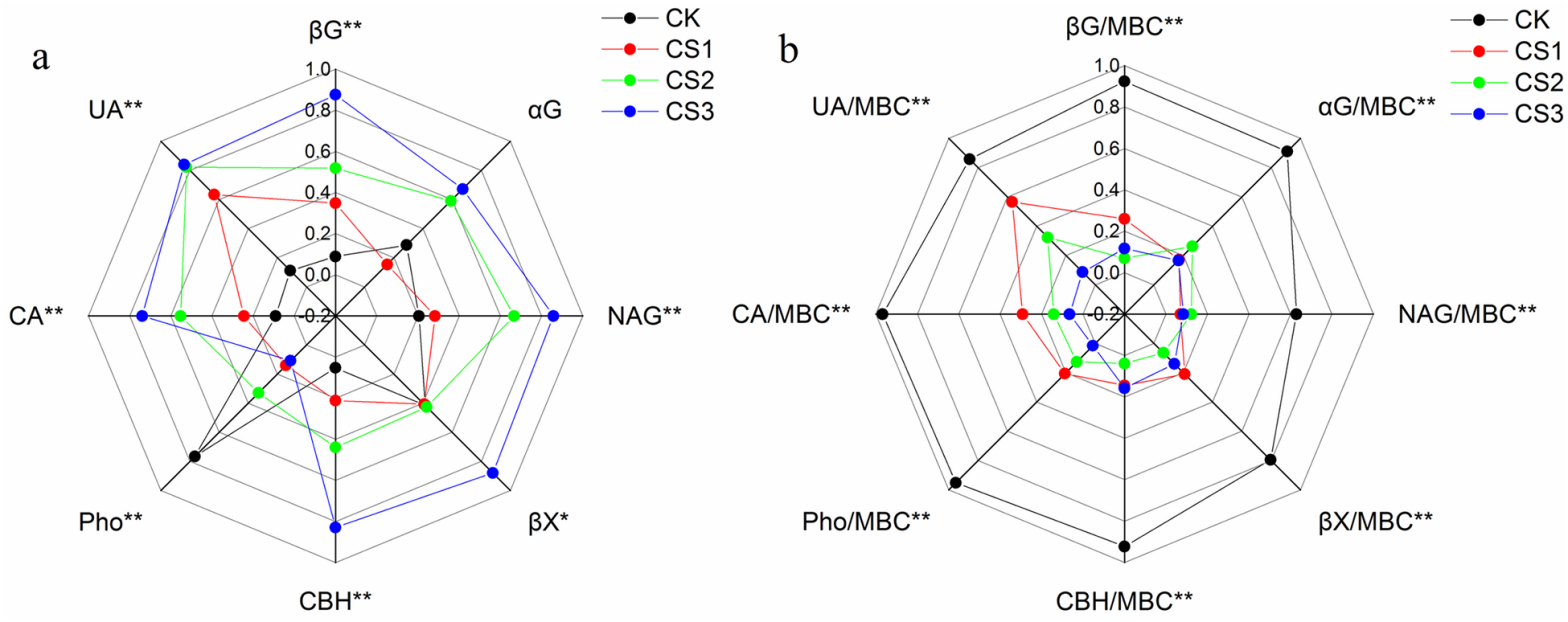
Radar charts of the relative response of absolute enzyme activity (a) and specific enzyme activity (b) to application of composted sewage sludge (CS). Significant differences are shown by asterisks among the different treatments (*, *P* <0.05; **, *P* <0.01; Fisher’s LSD test).

The activity of each of the eight soil enzymes related to C, N, and P cycling is shown in Figs 1 and 2a. The absolute activities of all enzymes except Pho and αG at all the three doses of CS were higher than those in CK. The activity of Pho was significantly decreased, by 17.5%–26.3%, in CS-treated soils, especially in CS3. Soil amendment with CS increased βG activity by 24.52%–74.29% and CBH activity by 17.65%–86.27%, the increase being directly proportional to the dose of CS, whereas βX activity was increased only in CS3, by 38.34%. Both CS2 and CS3 led to significantly increased activity of NAG (by 51.74%–59.26%), UA (by 57.32%–132.48%), and CA (by 22.70%–22.79%), although the differences between CS2 and CS3 were not significant. All the above values are as compared to those in CK and significant at 1%, except in the case of αG and βX.

However, the effect of CS on specific enzyme activity (Fig 2b) was different from that on absolute enzyme activity: the highest specific activity of all the enzymes was recorded in CK, whereas that in CS-treated soils was lower than CK, especially in CS3. Soil amended with CS recorded a significant decrease in αG, NAG, βX, and CBH, by 45.94%–52.69%, 40.94%–69.32%, 46.77%–58.33%, and 35.47%–41.02%, respectively, although the differences among the three doses were not significant. Specific enzyme activity of βG, Pho, CA, and UA decreased significantly, by 32.45%–41.80%, 59.18%–74.43%, 41.31%–51.19%, and 14.13%–37.54%, respectively, in CS-treated soils, especially in CS3. All the above values are as compared to those in CK and significant at 1%.

### Diversity of soil microbial function and community composition

**Fig 3:**
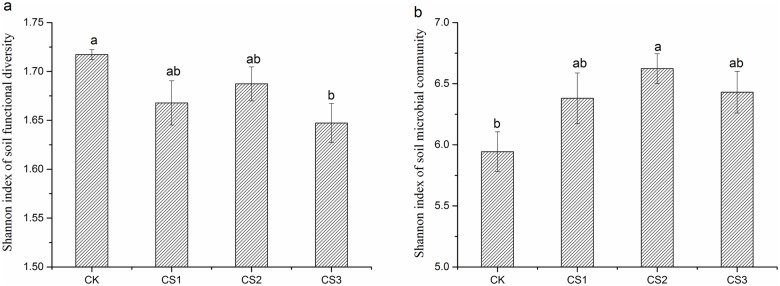
Shannon index of absolute enzyme activity (a) and soil microbial community (b) in soil under different treatments. Error bars represent standard error. Different letters indicate significant differences between different treatments (Fisher’s LSD test).

Shannon index of absolute enzyme activity decreased significantly only in CS3 (in CS2 and CS2, the decrease was not statistically significant) (Fig 3a), whereas Shannon index of microbial community composition increased significantly after the application of CS (Fig 3b).

### Factors influencing absolute and specific enzyme activity

**Fig 4:**
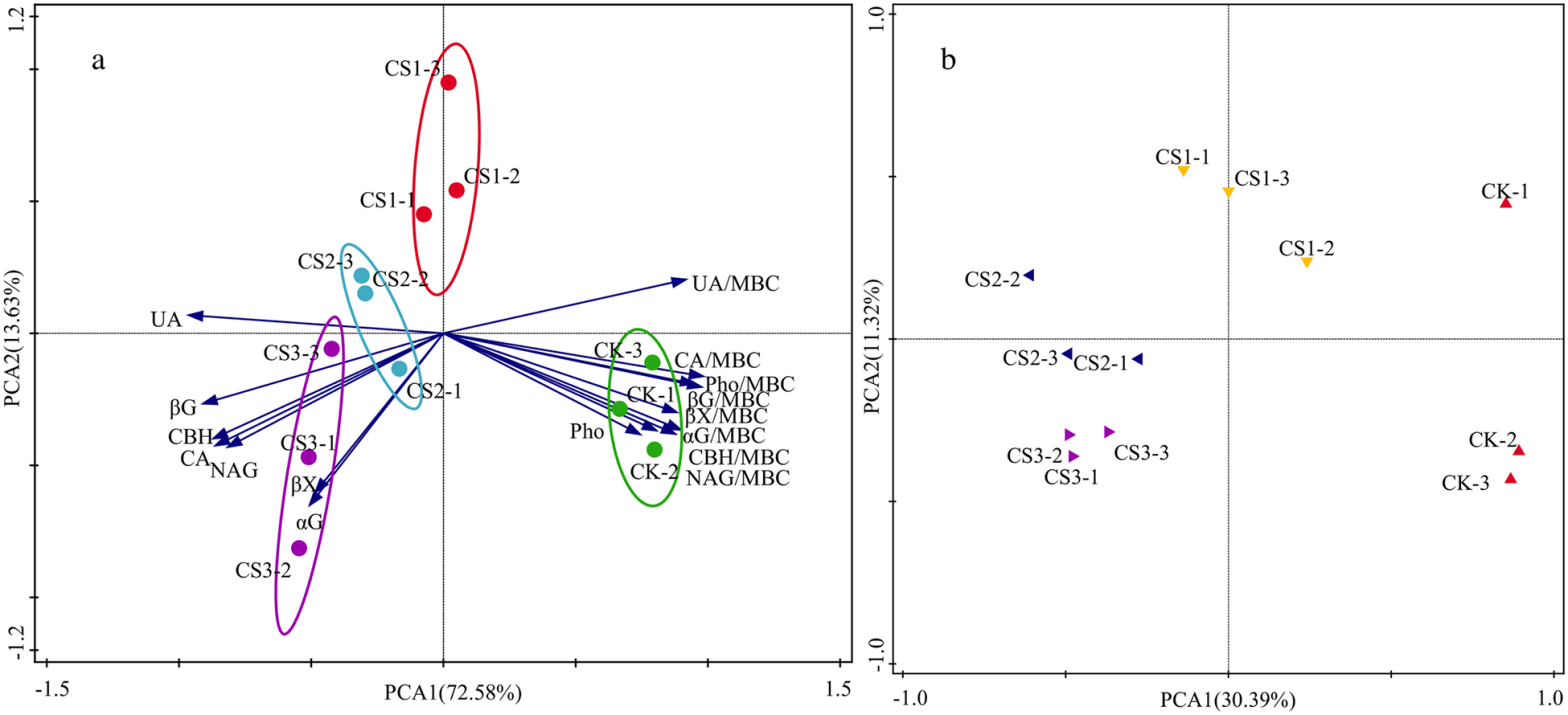
Principal component analysis (PCA) of absolute and specific enzyme activities (a) and operational taxonomic units (OTUs) obtained from soil (b) from different treatments. The first two axes represent percentage of most-explained variance.

Application of CS for three consecutive years led to pronounced differences between CS treatments and CK (Fig 4a), as shown by PCA: PCA1 was 72.91% and PCA2 was 13.15%. The dose of CS can represent PCA1, which separated the samples into four distinct groups. Each group had a specific pH. The highest pH (8.90) was recorded in CK, followed, in that order, by CS1 (8.56), CS2 (8.40), and CS3 (8.07). These results show that enzyme activity was influenced by changes in pH related to long-term addition of CS.

**Fig 5:**
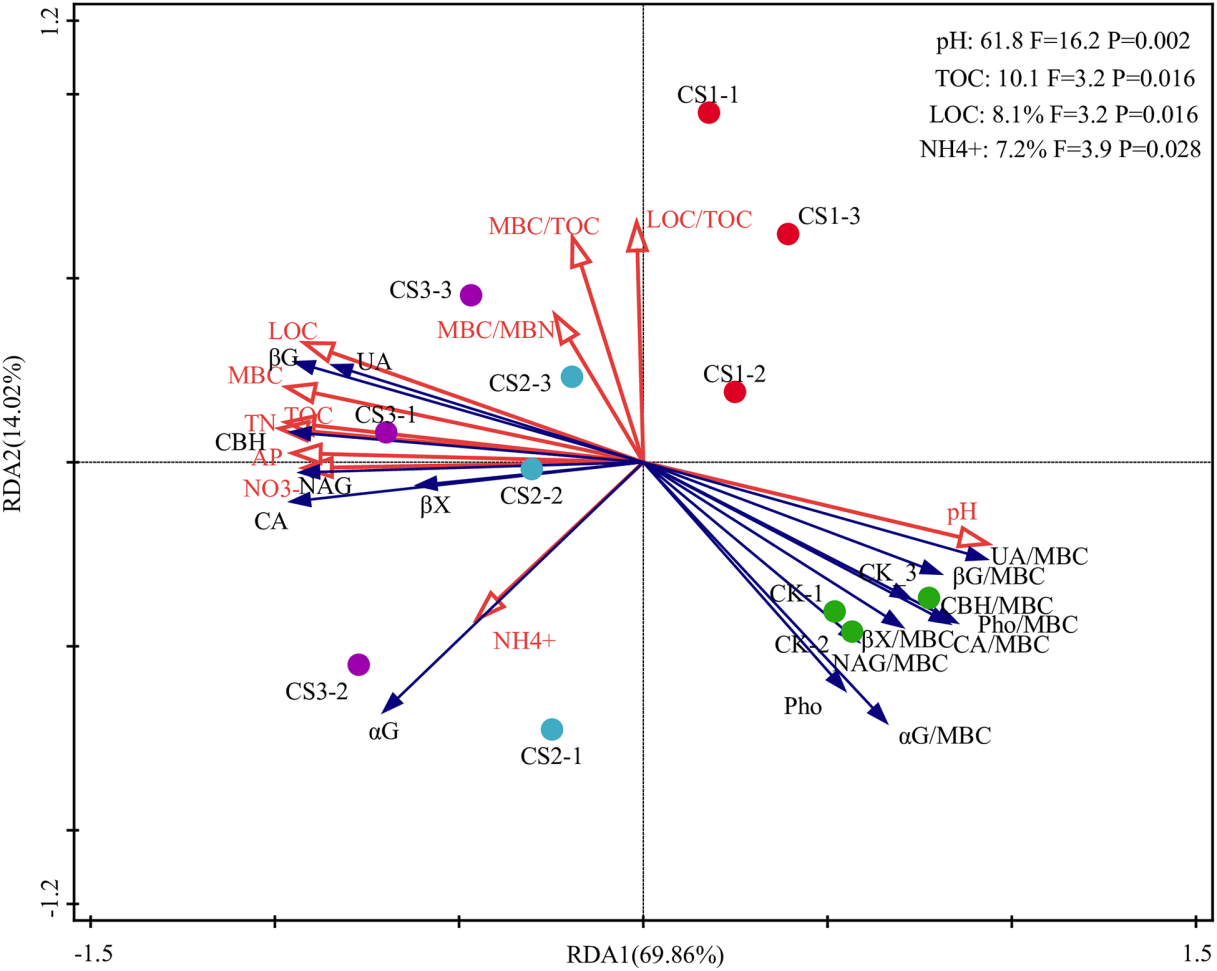
Redundancy analysis (RDA) of relationship between soil physicochemical properties and soil absolute and specific enzyme activities.

In RDA, the physicochemical properties of soil explained 96.16% of the variance in enzyme activity (Fig 5). The first two axes accounted for 69.33% and 13.20% of the variance, respectively. The analysis showed that CS-treated soils were clearly different from CK and CS-treated soils and also differed among themselves. These differences were mainly related to the alterations in pH, TOC, LOC, and NH_4_^+^, which explained 61.8% (*P* <0.01), 10.1% (*P* <0.05), 8.1% (*P* <0.05), and 7.2% (*P* <0.05) of the total variation as revealed by the interactive forward selection of RDA (Fig 5a). In descending order of influence, the sequence was as follows: pH, TOC, LOC, NH_4_^+^, AP, MBC, TN, NO_3_^−^, MBC/TOC, LOC/TOC, and MBC/MBN.

Pearson’s correlation coefficient was calculated for evaluating the relationship between enzyme activities (absolute and specific enzyme activities) and environmental factors (Table A in S1 Text). Both absolute enzyme activity and specific enzyme activity of all enzymes were significantly correlated to pH, TOC, TN, NO_3_^−^, AP, and MBC but there was no significant correlation between absolute and specific enzyme activities and NH_4_^+^. Specific enzyme activities were significantly correlated to MBC/TOC, LOC/TOC, and MBC/MBN, but absolute enzyme activities were not.

The highest dose of CS (CS3) significantly increased the concentrations in soil of all heavy metals detected in the present experiment, and those of Cu, Zn, Cd, and Cr increased even at the lower doses (Table 4). However, the changes were negligible in absolute terms and did not lower the quality of soil, because none of the heavy metals exceeded its maximum permissible concentration laid down by the CHN legislation (GB15918-1995).

**Table 4:** Concentrations of heavy metals in soil after consecutive application of CS during 2012–2015.

	Total Cu (mg kg^-1^)	Total Zn (mg kg^-1^)	Total Cd (mg kg^-1^)	Total Cr (mg kg^-1^)	Total Ni (mg kg^-1^)	Total Pd (mg kg^-1^)
CK	15.8±0.60c	49.9±1.50c	0.18±0.01d	36.9±0.37c	18.1±0.29b	14.84±0.12b
CS1	23.8±1.33b	67.8±1.29b	0.22±0.01a	44.4±2.11b	19.64±0.57ab	15.58±0.21ab
CS2	30.8±1.26b	95.8±3.01a	0.24±0.01a	50.7±1.61a	21.34±0.51b	16.04±0.33a
CS3	34.7±1.94a	94.1±5.19a	0.25±0.01a	54.76±0.86a	20.69±0.71a	15.91±0.35a
GB15618-1995	100	300	0.6	250	60	350

Values are mean ± standard deviation (N = 3). Different letters indicated significant differences in the same column (*P* <0.05, Fisher’s LSD test).

## Discussion

### Effects of annual application of composed sewage sludge for three years on physicochemical properties of soil

The significant increase in TOC, TN, NO_3_^−^, and AP in CS-treated soils, especially CS3, was directly related to the continued application of CS for 3 years and so were the high contents of C, N, and P [5]. The reduction in pH can be attributed to organic acids formed during the decomposition of CS, as reported earlier [27–28], and the smaller differences in NH_4_^+^ can be attributed to its oxidization to NO_3_^−^ in arid lands. The increase in NO_3_^−^ was due to the mineralization of N, which is also improved by the application of CS [4].

The higher values of MBC/MBN (significantly higher in CS1 and CS2 than those in CK) can also be attributed to the application of CS and can account for the different contents of C and N in bacteria and fungi: the value of MBC/MBN is generally 3–6 for bacteria but the range for fungi (5–15) is known to be much wider [29]. The increase in MBC/MBN in CS-treated soils showed that the composition of the soil microbial community had changed in favour of fungi to become a community dominated by fungi. Many studies have revealed that low pH favours the growth of fungi [30]. The increased predominance of fungi following the amendment with CS may be related not only to their preference for low pH, which is significantly lower in CS-treated soils, but also to their feeding behaviour, as they mainly decompose recalcitrant organic matter in soils [31]. Enowashu et al. (2009) [32] also found that any improvement in stocks of soil organic matter increases the biomass of fungi. It was found that CS contains high concentrations of hemicellulose, cellulose, and lignin [33], which are probably used by fungi as nutrients.

Microbial biomass carbon is regarded as a more accurate indicator than TOC of changes in soil conditions [34]. The significant increase in MBC was positively correlated to the dose of CS mainly because of the microbial biomass in CS and the addition of C substrate, which stimulated the native soil microflora, as pointed out by an earlier analysis [33]. However, several other researchers have reported a decrease in MBC owing to the high contents of heavy metals after the application of CS [35–36]. It was observed that the addition of such CS did not have inhibitory effects on MBC up to the studied does of CS because the quantities of heavy metals incorporated into the soil were small (Table 4). The same pattern was repeated for LOC: the significant increase in LOC was related to aerobically active particles of CS attached onto soil particles. However, it is difficult to assess the ecological status of a given environment simply by considering the size of its microbial pool (i.e. MBC content) [37]—other parameters related to microbial activity in soil (MBC/TOC) should also be considered. At lower doses (CS1 and CS2), CS increased the MBC/TOC because of the quality and quantity of organic matter it added to the soil, as confirmed by the significant increase in the LOC/TOC. However, higher doses of CS had a negative effect on the MBC/TOC. Jorge-Mardomingo et al. (2013) [16] and Fernández et al. (2005) [5] also found an improvement in MBC in CS-treated soils, and the lowest MBC/TOC was found at the highest doses of CS. Fernández et al. [5] (2005) suggested that the decrease in MBC/TOC could be attributed to three factors: (1) pH, (2) electrical conductivity (EC), and (3) heavy metals; however, none of these was a factor in the present study because no great variation in pH and EC was observed in CS-treated soils. According to a number of studies [38–39], a high concentration of heavy metals could also result in the reduction of MBC/TOC. Brookes (1995) [40] also used MBC/TOC as an index of soil pollution by heavy metals, but in the present study, concentrations of heavy metals even in CS-treated soils were fairly low (Table 4)—far below the limits proposed by the CHN legislation (GB15918-1995). Tate (1987) [41] reported that the decrease in MBC/TOC at high doses of CS could be due to a high degree of condensation and humification of soil organic matter, which make the organic matter resistant to breakdown by microbes. The highest proportion of recalcitrant organic carbon in CS3, confirmed by the lowest LOC/TOC, could have decreased the MBC/TOC. Moreover, according to Anderson and Domsch (2010) [42], the entire demand for maintaining cell growth when carbon is not a limiting factor differs from that in a natural environment where carbon is limited, as in the semi-arid ecosystem represented in the present study. A high content of LOC, an improvement in EC, and a decrease in pH would be caused by higher doses of CS [28]. These results could have resulted in ecological pressure on soil microbial community, leading to greater demands of energy for growth and, in turn, inhibition of microbial activity.

### Effect of application of composted sewage sludge on absolute enzyme and specific enzyme activities

Soil enzyme activities play a key role in the degradation of soil organic matter and nutrient cycling [8]. The highest significant increase in the activities of enzymes—except αG—involved in the C cycle (Figs 1 and 2a) was observed in CS3. Of these enzymes, βG mainly works on cellulose and degrades short-chain oligosaccharides and cellobiose; CBH catalyses the hydrolysis of cellulose; βX catalyses the hydrolysis of hemicellulose; and NAG is important in the cycling of both C and N in soil. From the value of LOC, it can be inferred that CS-treated soils were richer in cellulose, sugars, carbohydrates, and hemicellulose—their contents in soil correlated well with their corresponding amounts in CS—and probably stimulated the microorganisms to produce more of βG, CBH, and βX. Chuang et al. (1994) [43] reported that fungi are an important source of chitin. The significant increase in NAG in CS-treated soils was related to the greater availability of chitin originating from the biomass of dead fungi.

The activity of UA, the enzyme related to the N cycle, improved remarkably in CS-treated soils, especially in CS3, because CS was rich in organic N. Earlier studies reported low activity of UA in CS-treated soils and attributed it to high contents of NH_4_^+^, which could inhibit UA [16]. However, NH_4_^+^ content was low in CS-treated soils in the present experiment and therefore no inhibition was observed.

The opposite pattern was observed in the P cycle: the activity of Pho declined with increasing dose of CS and was the highest in CK. Similar results were reported by other researchers [44–45] and were related to the retroaction inhibition by inorganic phosphates present in large amounts in CS-treated soils. As to CA, the enzyme is usually associated with aerobic microorganisms [46] and its activity was greater in CS-treated soils and was positively correlated to that of other enzymes except Pho and βX (Table B in S1 Text). Consequently, the mineralization of CS increased the amount of substrate for βG and for microbial growth and therefore CA was enhanced owing to this process, as García-Gil et al. (2000) [47] reported.

In contrast, the highest specific enzyme activity was observed in CK, and overall specific enzyme activity showed lower values in CS-treated soils, especially in CS3 (Fig 2b). The indigenous microorganisms in the soil adjusted better to the carbon-limited environment. Therefore, higher specific enzyme activities were recorded in CK. The lower value of specific enzyme activity in CS-treated soils could imply decreasing enzyme production and release by microorganisms. Specific activity of every enzyme was significantly correlated to that of every other enzyme (Table C in S1 Text), indicating that all specific enzyme activities are closely interrelated and the activity of any one enzyme affects the activity of all the other enzymes significantly.

A larger microbial pool (MBC and MBC/TOC) in CS-treated soils than that in CK corresponded to lower specific enzyme activity. This observation implies domination of microorganisms that are not metabolically active and those that use resources more efficiently, namely fungi, probably due to the quality and quantity of available organic matter and the characteristics of CS. This hypothesis is strongly supported by the improvement in MBC/MBN described above. Moreover, it could also be confirmed by the improvement in NAG, which is an indirect indicator of the biomass of soil fungi [9].

### Responses of soil functional diversity and soil microbial community diversity

Degens et al. (2000) [48] showed a generalized relationship between the pool of organic C and the diversity of microbial functions in soil. In the present study, soils under different doses of CS were functionally different and all the treatments were distinct from each other because of the quantity, quality, and accessibility of substrates (Fig 4a). Shannon index of soil microbial functional diversity showed that the largest microbial functionality was found in CK and that it decreased significantly in CS3 (Fig 3a). The result was related to significantly lowered specific enzyme activity in CS-treated soils, especially in CS3. Therefore, specific enzyme activity was more closely related to the soil microbial functional diversity than absolute enzyme activity, as reported by Waldrop et al. (2000) [49].

It was found that extracellular enzymes were connected with living microbial cells and abiotic ingredients and could not represent the whole soil microbial activity [50]. Therefore, we used high-throughput sequencing to evaluate the overall diversity of microbial community. In PCA of microbial community composition, the treatments were also clearly separated from each other, indicating that adding CS had changed the overall microbial diversity and that different doses of CS had different effects on the overall microbial diversity (Fig 4b). Shannon index of microbial community composition revealed higher overall microbial diversity in CS-treated soils than that in CK and lower overall microbial diversity in CS3 compared to that in CS1 and CS2 (Fig 3b): both observations probably reflect the higher values of MBC/TOC and LOC/TOC in CS-treated soils. The highest dose of CS led to humification of soil organic matter and acted as a source of ecological stress for soil microorganisms as described above, which had a negative effect on microbial diversity.

High overall microbial diversity corresponded to lower functional performance of the microbial community in CS-treated soils, especially CS3, probably because of the predominance of microorganisms that are not metabolically active and of those organisms that use resources more efficiently, namely fungi-a result that provides additional evidence in support of the hypothesis described above.

### Responses of absolute enzyme activity to alterations in soil physicochemical properties

Both PCA and RDA showed that fundamental changes in absolute and specific enzyme activities depended on soil pH (Figs 4a and 5). Other studies have also found that soil amendments affect absolute enzyme activity mainly through their influence on soil pH [51]. Absolute enzyme activities were higher in soil with pH closer to its neutral value (pH of 7). The increased absolute enzyme activity accelerated the degradation of organic matter, and such enzyme-induced improvement in soil nutrients may be an important mechanism to compensate for the low nutrient status of Fluventic Ustochrept. Certainly, it was observed that soil nutrients were remarkably and positively correlated to absolute activities of all enzymes except Pho, which was negatively correlated to soil nutrients (Table A in S1 Text). Soil nutrients were also significantly and negatively correlated to the specific activity of all enzymes (Table A in S1 Text), which suggests that higher content of nutrients and lower pH could inhibit the release of enzymes by microorganisms. Specific enzyme activities were also significantly and negatively correlated to MBC/TOC, LOC/TOC, and MBC/MBN, whereas absolute enzyme activities showed no significant correlation to the three ratios (Table A in S1 Text). These differences suggest that specific enzyme activity is a better indicator of changes in soil microbial activity and in microbial community composition.

## Conclusion

Addition of CS to soil brought about desirable changes in the physicochemical properties of soil and increased absolute activity of all enzymes. Soil microbial functional diversity decreased as the dose of CS increased, which resulted from the decrease in specific enzyme activity. On the other hand, the composition of the soil microbial community became more diverse in CS-treated soils, suggesting domination of microorganisms that are not metabolically active and those that use resources more efficiently, namely fungi. Thus, an assay of soil absolute enzyme activity combined with MBC can serve as a more sensitive indicator of the effect of applying CS to Fluventic Ustochrept. Both PCA and RDA revealed that fundamental changes in soil enzyme activity depended on soil pH, and the Pearson correlation coefficient between enzyme activity and soil properties also showed that specific enzyme activity in soil is more closely affected by soil properties than absolute enzyme activity. In general, specific enzyme activity proved a clearer and more useful indicator in detecting and assessing alterations in the physicochemical properties of soil and in the metabolic status of the soil microbial community following CS application to Fluventic Ustochrept.

## Supporting information

S1 Dataset Soil absolute enzyme activity and specific enzyme activity under CK, CS1, CS2 and CS3 treatments. (XLSX)

S1 Text This text includes three tables: Correlation between enzyme activity and soil physicochemical properties (Table A in S1 Text), correlation among eight absolute enzyme activities (Table B in S1 Text), and correlation among eight specific enzyme activities (Table C in S1 Text). (DOCX)

## Abbreviations

AP: available phosphorus
CS: composted sewage sludge
LOC: labile organic carbon
MBC: microbial biomass carbon
MBN: microbial biomass nitrogen
TN: total nitrogen
TOC: total organic carbon
