# Supplementary material for: Responses of absolute and specific enzyme activity to consecutive application of composted sewage sludge in a Fluventic Ustochrept
Source: PLoS One. 2017 May 17;12(5):e0177796. doi: 10.1371/journal.pone.0177796 (PMC5435317; doi:10.1371/journal.pone.0177796)
Supplement: S1 Text — (DOCX) [file pone.0177796.s002.docx]

**Table A** Correlation between enzyme activity and soil physicochemical properties.

|  | pH | TOC | TN | NH4+ | NO_3_^-^ | AP | MBC | LOC | MBC/TOC | LOC/TOC | MBC/MBN |
| --- | --- | --- | --- | --- | --- | --- | --- | --- | --- | --- | --- |
| βG | -0.985** | 0.973** | 0.917** | 0.29 | 0.821** | 0.865** | 0.946** | 0.917** | 0.167 | 0.038 | 0.208 |
| αG | -0.53 | 0.657* | 0.652** | 0.584* | 0.722** | 0.719** | 0.579* | 0.491 | -0.189 | -0.292 | -0.047 |
| NAG | -0.882** | 0.909** | 0.903** | 0.52 | 0.822** | 0.819** | 0.880** | 0.824** | 0.103 | -0.08 | 0.238 |
| βX | -0.569 | 0.663* | 0.497 | -0.167 | 0.494 | 0.573 | 0.565 | 0.460 | -0.247 | -0.389 | -0.068 |
| CBH | -0.891* | 0.951** | 0.874** | 0.313 | 0.838** | 0.894** | 0.927** | 0.852** | 0.139 | -0.073 | 0.05 |
| Pho | -0.581* | -0.600* | -0.616* | 0.14 | -0.536 | -0.561 | -0.667 | -0.786** | -0.574 | -0.665 | -0.42 |
| CA | -0.861** | 0.932** | 0.916** | 0.538 | 0.914** | 0.953** | 0.917** | 0.847** | 0.179 | -0.024 | 0.143 |
| UA | -0.839** | 0.856** | 0.932** | 0.423 | 0.924** | 0.937** | 0.925** | 0.939** | 0.588* | 0.427 | 0.506 |
| βG/MBC | 0.777** | -0.799** | -0.899** | -0.393 | -0.897** | -0.878** | -0.896** | -0.929** | -0.710** | -0.544 | -0.612* |
| αG/MBC | 0.771** | -0.703* | -0.761** | -0.126 | -0.669* | -0.698* | -0.809** | -0.869** | -0.721** | -0.598* | -0.598* |
| NAG/MBC | 0.598* | -0.639* | -0.688* | -0.136 | -0.714** | -0.700** | -0.729** | -0.783** | -0.658* | -0.567 | -0.485 |
| βX/MBC | 0.744** | -0.719** | -0.843** | -0.496 | -0.773** | -0.767** | -0.818** | -0.883** | -0.697* | -0.604* | -0.597* |
| CBH/MBC | 0.745** | -0.715** | -0.841** | -0.356 | -0.800** | -0.773** | -0.816** | -0.890** | -0.732** | -0.645* | -0.715** |
| Pho/MBC | 0.843** | -0.845** | -0.908** | -0.264 | -0.858** | -0.867** | -0.922** | -0.974** | -0.650* | -0.549 | -0.568 |
| CA/MBC | 0.877** | -0.863** | -0.928** | -0.310 | -0.870** | -0.877** | -0.939** | -0.974** | -0.635* | -0.491 | -0.577* |
| UA/MBC | 0.938** | -0.938** | -0.928** | -0.345 | -0.832** | -0.863** | -0.952** | -0.920** | -0.324 | -0.124 | -0.297 |

**Table B** Correlation among eight absolute enzyme activities

|  | βG | αG | NAG | βX | CBH | Pho | CA | UA |
| --- | --- | --- | --- | --- | --- | --- | --- | --- |
| βG | 1 |  |  |  |  |  |  |  |
| αG | 0.503 | 1 |  |  |  |  |  |  |
| NAG | 0.895** | 0.651* | 1 |  |  |  |  |  |
| βX | 0.657* | 0.442 | 0.537 | 1 |  |  |  |  |
| CBH | 0.913** | 0.627* | 0.853** | 0.707* | 1 |  |  |  |
| Pho | -0.626* | -0.079 | -0.431 | -0.241 | -0.514 | 1 |  |  |
| CA | 0.844** | 0.799** | 0.834** | 0.554 | 0.930** | -0.42 | 1 |  |
| UA | 0.788** | 0.551 | 0.701* | 0.392 | 0.777** | -0.664* | 0.861** | 1 |

Enzyme abbreviations: Pho phosphatase, CA catalase, βG β-glucosidase, CBH cellobiohydrolase, NAG N-acetylglucosaminidase, βX β-xylosidase, αG α-glucosidase, UA urease.

**Table C** Correlation among eight specific enzyme activities

|  | βG/MBC | αG/MBC | NAG/MBC | βX/MBC | CBH/MBC | | Pho/MBC | CA/MBC | UA/MBC |
| --- | --- | --- | --- | --- | --- | --- | --- | --- | --- |
| βG/MBC | 1 |  |  |  |  |  | |  |  |
| αG/MBC | 0.863** | 1 |  |  |  |  | |  |  |
| NAG/MBC | 0.860** | 0.826** | 1 |  |  |  | |  |  |
| βX/MBC | 0.909** | 0.861** | 0.717** | 1 |  |  | |  |  |
| CBH/MBC | 0.944** | 0.900** | 0.845** | 0.932** | 1 |  | |  |  |
| Pho/MBC | 0.969** | 0.922** | 0.862** | 0.907** | 0.950** | 1 | |  |  |
| CA/MBC | 0.963** | 0.937** | 0.833** | 0.910** | 0.951** | 0.991** | | 1 |  |
| UA/MBC | 0.782** | 0.782** | 0.543 | 0.768** | 0.725** | 0.844** | | 0.880**. | 1 |

Enzyme abbreviations: Pho phosphatase, CA catalase, βG β-glucosidase, CBH cellobiohydrolase, NAG N-acetylglucosaminidase, βX β-xylosidase, αG α-glucosidase, UA urease.
